# Photomechanical meta-molecule array for real-time terahertz imaging

**DOI:** 10.1038/micronano.2017.71

**Published:** 2017-12-04

**Authors:** Yongzheng Wen, Delin Jia, Wei Ma, Yun Feng, Ming Liu, Liquan Dong, Yuejin Zhao, Xiaomei Yu

**Affiliations:** 1 National Key Laboratory of Science and Technology on Micro/Nano Fabrication, Institute of Microelectronics, Peking University, Beijing 100871, China; 2 State Key Laboratory of New Ceramics and Fine Processing, School of Materials Science and Engineering, Tsinghua University, Beijing 100084, China; 3 Beijing Key Laboratory for Precision Optoelectronic Measurement Instrument and Technology, School of optoelectronic, Beijing Institute of Technology, Beijing 100081, China

**Keywords:** bi-material cantilever, meta-atom absorber, photomechanical meta-molecule array, polyimide sacrificial layer technique, terahertz imaging

## Abstract

Real-time terahertz (THz) imaging offers remarkable application possibilities, especially in the security and medical fields. However, most THz detectors work with scanners, and a long image acquisition time is required. Some thermal detectors can achieve real-time imaging by using a focal plane array but have the drawbacks of low sensitivity due to a lack of suitable absorbing materials. In this study, we propose a novel photomechanical meta-molecule array by conveniently assembling THz meta-atom absorbers and bi-material cantilevers together, which can couple THz radiation to a mechanical deflection of the meta-molecules with high efficiency. By optically reading out the mechanical deflections of all of the meta-molecules simultaneously, real-time THz imaging can be achieved. A polyimide sacrificial layer technique was developed to fabricate the device on a glass wafer, which facilitates the transmission of a readout light while the THz wave radiates onto the meta-molecule array directly from the front side. THz images and video of various objects as well as infrared images of the human body were captured successfully with the fabricated meta-molecule array. The proposed photomechanical device holds promise in applications in single and broadband THz as well as infrared imaging.

## Introduction

Terahertz (THz) imaging has received substantial attention and has become a highly active field over the past two decades, owing to the unique properties of THz waves, thus leading to promising applications in numerous fields, including security screening, medical imaging, and remote sensing^1–3^. A variety of commonly used packaging materials, such as paper, fabrics and plastic, are transparent to THz waves; this technology may thus have potential in the security field and non-destructive inspections^4,5^. The low photon energy of THz radiation causes no ionization risk, and the THz fingerprint spectra for biomolecules may allow THz imaging to be used as a tool for medical diagnoses and analyses, especially for skin burns and skin cancers^6,7^.

Accordingly, continuous efforts have resulted in the development of high-sensitive and real-time THz detectors for use as the key component in THz imaging systems^8^. Generally, THz detectors can be classified into two categories: photonic detectors and thermal detectors. Despite the high sensitivity and extremely fast response time, the sensible band of most photonic detectors is narrow, and their typical requirement of cryogenic cooling systems causes them to be expensive and bulky^9–11^. The thermal detector absorbs the THz radiation as heat, which generates measurable output signals induced by the temperature-related changes in material properties. A variety of thermal detectors are capable of operating at room temperature with decent sensitivity and response time, such as thermopiles, pyroelectric detectors and Golay cells^10,12,13^. Most available THz imaging systems operate in the scan mode by using the above-mentioned photonic or thermal detectors, which may require between tens of seconds and several hours to obtain one image^14,15^. More recently, a modified infrared microbolometer-based focal plane array has preliminarily been demonstrated in real-time THz imaging applications and requires only tens of microseconds to shoot one THz image^16^; however, its sensitivity suffers considerably because of the lack of suitable THz-absorbing materials.

Artificial metamaterials, with optical properties not normally found in nature, may be a promising solution, and they have already been used in a wide variety of applications, such as ultrathin polarizers, holograms, flat lenses, and perfect absorbers^17–20^. Because the performance of the metamaterial absorber is primarily determined by the designed structure of the meta-atom rather than its composites, the microfabrication friendly materials, typically demonstrate low loss in the THz regime, can convert the THz energy to heat with high efficiency. Recently, several researchers have attempted to combine the metamaterial with micromechanical structures^21^. By mechanically changing the geometry of the metamaterials, the optical properties can be modulated effectively, and several novel phenomena have been realized, such as state switching, anisotropy control, and resonance shifting^22–24^. However, most studies have focused on realizing actively tunable metamaterials, and the novel properties of photomechanical metamaterials, which may have numerous potential applications, for example, THz imaging, have been relatively less studied.

In this study, we propose a photomechanical meta-molecule array containing 128×128 pixels for active real-time THz imaging. By assembling the meta-atom absorber with thermal-sensitive bi-material cantilevers, the photomechanical meta-molecule array can image the THz wave by turning its photonic energy into mechanical deformation through heat, which can then be optically read out. The meta-molecule array was fabricated on a glass wafer, which facilitates the transmission of the readout light while the THz wave directly illuminates the array from the front side. Additionally, this innovative design avoids THz energy loss from the substrate. An active THz imaging system was set up to acquire THz images and video of different objects by using the fabricated meta-molecule array at room temperature. Furthermore, the SiN_x_ layer in the meta-atom absorber allows for passive infrared imaging.

## Materials and methods

### Design of the photomechanical meta-molecule array

The most critical characteristic of the meta-molecule is responsivity, *R_v_*, which is defined as the displacement of the meta-molecule tip, Δ*z*, under a given incident THz radiation power falling on one meta-molecule, *P*_0_, and it can be expressed as follows^25^:

(1)Rv=ΔzP0=ηSTGtotal1+ω2τ2,

where *S_T_* is the thermomechanical sensitivity; *η* is the THz absorptivity of the meta-atom absorber; *ω* is the modulation frequency of the incident THz wave; *G*_total_ is the total thermal conductance of the meta-molecule; and *τ* is the response time. It can be determined from Equation (1) that a high thermomechanical sensitivity and THz absorptivity and a low total thermal conductance are all beneficial for reaching a high responsivity. Because the thermomechanical sensitivity presents the relation of *S_T_∝L*_bi_^2^*×(α*_1_−*α*_2_), where *L*_bi_ is the length of the bi-material cantilever and *α*_1_ and *α*_2_ are the thermal coefficients of expansion (TCE) for the two materials, it can be seen that a large TCE difference and a long bi-material cantilever length would increase the thermomechanical sensitivity. Furthermore, the implementation of the thermal isolation cantilever is required to minimize the total thermal conductance. The detailed design theory is described in the Supplementary Information.

**Figure 1:**
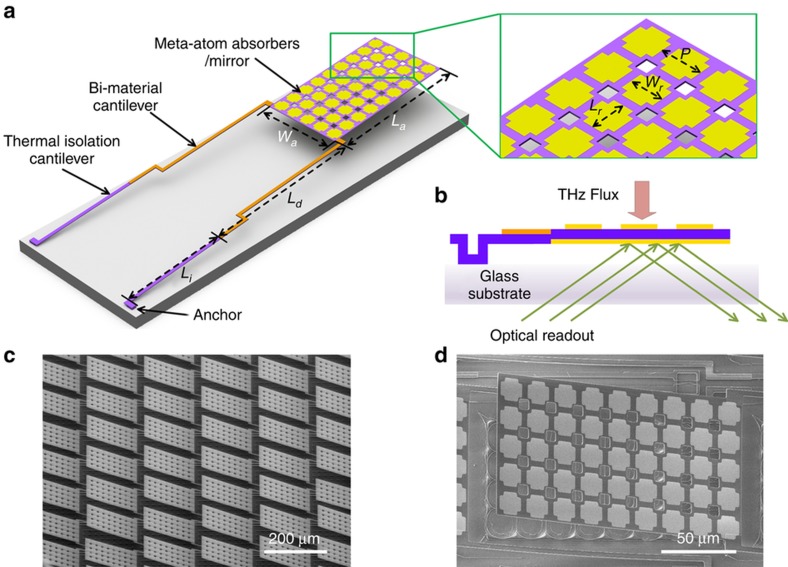
Overview of the photomechanical meta-molecule array. Perspective (**a**) and side (**b**) views of one meta-molecule containing a meta-atom absorber, two bi-material cantilevers, thermal isolation cantilevers and anchors. The dimensions of the meta-molecule are as follows: *L_a_*=180 μm; *W_a_*=100 μm; *L_d_*=209 μm; *L_i_*=133 μm; *L_r_*=16 μm; *W_r_*=10 μm; and *P*=20 μm. Scanning electron microscope (SEM) images of the fabricated array (**c**) and one meta-molecule (**d**).

The schematic diagram of one meta-molecule and a fabricated photomechanical meta-molecules array containing 128×128 pixels is depicted in Figure 1. Each meta-molecule consists of a meta-atom absorber with a high THz absorptivity suspended by two symmetric cantilevers anchored on an optically transparent substrate. Each cantilever is segmented into a bi-material cantilever and a thermal isolation cantilever. The bi-material cantilever consists of stacked layers of 400 nm-thick Al on 800 nm-thick SiN_x_, and their thermal coefficients of expansion differ by two orders of magnitude^26^, thus resulting in a distinct deflection with a mild temperature shift due to the thermal mismatch between the two composite materials, thus guaranteeing a high thermomechanical sensitivity. The thermal isolation cantilever consists of a single 800 nm-thick SiN_x_ layer with a low thermal conductivity, which can minimize the heat dissipation from the meta-molecule to the substrate and reduce the total thermal conductance.

The meta-atom absorber, the key element that enables the photomechanical meta-molecule array to sense the THz wave with high sensitivity, was designed by using a simple sandwiched structure. In the structure, a 50 nm-thick gold ground plane and top THz resonators were separated by an 800 nm-thick SiN_x_ dielectric spacer. A proper pattern on the THz resonators allowed the sandwiched structure to resonate with the incident THz wave and serve as a THz absorber. Polarization-independent cross-shaped resonators were used and embedded between releasing holes, which have no noticeable influence on the absorption performance^27^. The gold ground plane backing the meta-atom absorber served as a reflective mirror for both the THz wave and the visible light of the optical readout, and the SiN_x_ spacer was used as the structural layer of the meta-molecule. Therefore, a simple assembly of the meta-atom absorber with the bi-material cantilever formed the meta-molecule. The geometric parameters of the entire meta-molecule were optimized by considering the absorption efficiency and mechanical sensitivity of the device as well as the ease of fabrication (Supplementary Information).

When the THz flux radiates onto the meta-molecule array from the front side, the meta-atom absorbers transform the THz radiation into heat, and the bi-material cantilever causes a mechanical deflection to the meta-molecule, which is then read out by a collimated beam of visible light incident on the reflective mirror through the transparent substrate. This approach results in no THz energy loss from the substrate. Because the deflections of all of the meta-molecules in the array are acquired simultaneously, real-time THz imaging can be achieved. Additionally, because the photomechanical meta-molecule is a non-electric approach, the array scale can be easily extended to high-resolution imaging of the THz wave.

### Microfabrication of the photomechanical meta-molecule array

**Figure 2:**
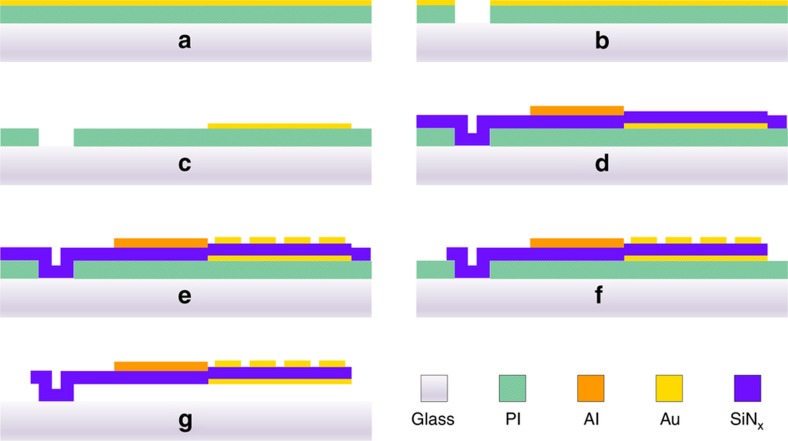
Microfabrication processes for the photomechanical meta-molecule array. (**a**) spin coating of PI and sputtering of Au/Ti on the glass wafer; (**b**) the anchors were patterned by dry etching PI using the Au/Ti as the hard mask; (**c**) the Au/Ti mirror was patterned and wet etched as the mirror; (**d**) after the deposition of SiN_x_, an Al layer of the bi-material cantilevers was sputtered and wet etched; (**e**) a Ti/Au layer was evaporated while the top resonators were patterned; (**f**) meta-molecule structures were defined by dry etching the SiN_x_ layer; and (**g**) the device was released with the isotropic dry etching of PI.

The meta-molecule array was fabricated by using a polyimide (PI) sacrificial layer technique based on the fabrication processes illustrated in Figure 2. A 400 μm-thick glass wafer was used as the transparent substrate for the optical readout. By considering the process compatibility and avoiding the stiction caused by wet releasing, a 1.8 μm-thick PI was spin coated on the substrate as the sacrificial layer. Then, a 50/20 nm-thick Au/Ti was sputtered and wet etched as the hard mask to define the anchors, and this was followed by an anisotropic dry etching of the PI layer in oxygen plasma. The Ti film on top served as the adhesion layer between Au and the SiN_x_ spacer. In the third step, the above Au/Ti film was patterned again as the ground plane/mirror. In the fourth step, an 800 nm-thick SiN_x_ spacer was deposited by using the plasma enhanced chemical vapor deposition (PECVD) technique, a low temperature process compatible with the melting temperature of the PI. Then, another 400 nm-thick Al film was sputtered and defined as the top metal layer of the bi-material cantilever by using wet etching. Afterwards, a 20/50 nm-thick Ti/Au was e-beam evaporated and patterned by using a lift-off process to form the top cross resonators of the meta-atom absorbers. Then, the SiN_x_ layer was patterned as the overall structure of the meta-molecules by using reactive ion etching. Finally, the meta-molecule array was completely released by the isotropic dry etching of the PI sacrificial layer with an oxygen plasma asher. Figures 1c and d depict the scanning electron microscope (SEM) images of the fabricated meta-molecule array and one meta-molecule, respectively.

### Setup of the imaging system

**Figure 3:**
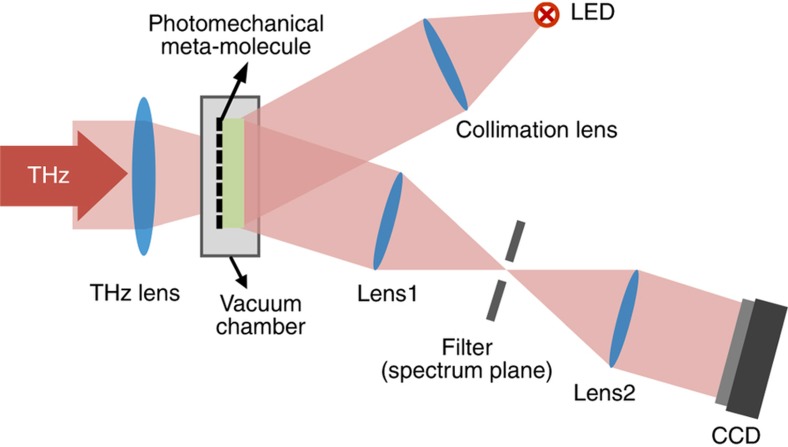
Schematic of the real-time imaging system. All components are labeled, and the photomechanical meta-molecule array is sealed in a vacuum chamber.

The schematic diagram of the imaging system is depicted in Figure 3. The system operated at room temperature without requiring any cryogenic equipment. To prevent heat dissipation through air, the photomechanical meta-molecule array was sealed in a vacuum chamber with an operating pressure <0.1 Pa. An optically pumped THz laser (OPTL) radiating 3.11 THz continuous wave was used as the source for the active THz imaging, and the average THz power reaching the meta-molecule array was measured to be 6.9 mW by using a THz power meter (Ophir Nova II with 3A-P-THz). The material used for the THz lenses and window of the vacuum chamber was high-density polyethylene (HDPE), thus providing a high transmission rate for the THz wave^28^. When functioning, the THz radiation from the objects was gathered by the lenses and illuminated on the meta-molecule array through the incident window while a beam of collimated light from a light-emitting diode (LED) was cast on the mirrors of the meta-molecules through the readout window. The reflected light from the mirror was filtered by a knife-edge at the spectrum plane, which was then probed by an 8-bit CCD. Finally, the data of the images were collected and processed with a computer.

### Simulations and characterizations of the photomechanical meta-molecule

The reflection spectrum, *R*(*ω*), in the THz regime was simulated by using a finite element package (COMSOL Multiphysics). Because the thickness of the Au ground plane was considerably thicker than its skin depth in the THz regime and most transmission is blocked, the absorption spectrum, *A*(*ω*), can be simply calculated by *A*(*ω*)*=1−R*(*ω*). Au was specified to have a conductivity of 1.8×10^7^ S m^−1^, which was measured by using a four-probe meter, and a permittivity of 7.5, where a loss tangent of 0.025 was applied to SiN_x_.

The THz reflection spectrum of the meta-atom absorber was measured using a Fourier transform infrared (FTIR) spectrometer (Bruker IFS125HR) extended to THz range with a mercury vapor source and a 50 μm Mylar beam splitter. The experiment was performed in a vacuum to minimize the atmospheric loss with a thick gold mirror as a reference.

Noise equivalent power (NEP) is a common parameter used to evaluate the sensitivity of the THz detector, and it can be defined as the signal power that results in a signal-to-noise ratio of one in a one hertz output bandwidth^29^. By definition, the NEP of the imaging system can be expressed as NEP=*z_n_/R_v_* (Refs. 25,30,31), where *z_n_* is the displacement of the meta-molecule tip caused by the total noise. As stated in Equation (1), the responsivity can be defined as *R_v_*=*z*_0_*/P*_0_. Because the displacement of the meta-molecule dip is proportional to the greyscale level of the image captured by the CCD^31^, the NEP can be calculated as NEP=*P*_0_·(*G_n_/G*_0_), where *G_n_* and *G*_0_ are the greyscale levels of the noise and signal images, respectively, and *P*_0_ is the THz power falling on one meta-molecule. In the measurement of the NEP, the noise image was acquired by recording the output of the CCD without the THz radiation, whereas the signal image was recorded by the CCD with the THz laser radiating on the array. Therefore, with the known incident power of the THz wave, the NEP can be extracted.

The response time, *τ*, is a critical parameter used to estimate the real-time response of a detector, which can be generally defined as the time required for a transient output signal to reach 1−1/*e* (≈0.632) of its steady-state change. We used COMSOL Multiphysics to simulate the response time of the meta-molecule. The thermal conductivities and specific heat capacities of Au, SiN_x_ and Al used in the model are listed in Supplementary Table S1. By examining the change in the transient temperature of the meta-molecule in the time domain with a loaded heat flux of 0.5 W m^−2^, the response was thus obtained. During the measurement, a metal shutter was placed between the OPTL and the photomechanical meta-molecule array to switch the THz wave on and off in less than 5 ms. A high speed CCD (Baumer HXC20NIR) was used to record the transient greyscale in the time domain with a frame rate of 250 Hz corresponding to the time step of 4 ms. Therefore, the experimental value of the response time was extracted from the time-domain greyscale change curve, on the basis of its proportional relationship with the meta-molecule temperature.

## Results and discussion

### Characteristics of the photomechanical meta-molecule

**Figure 4:**
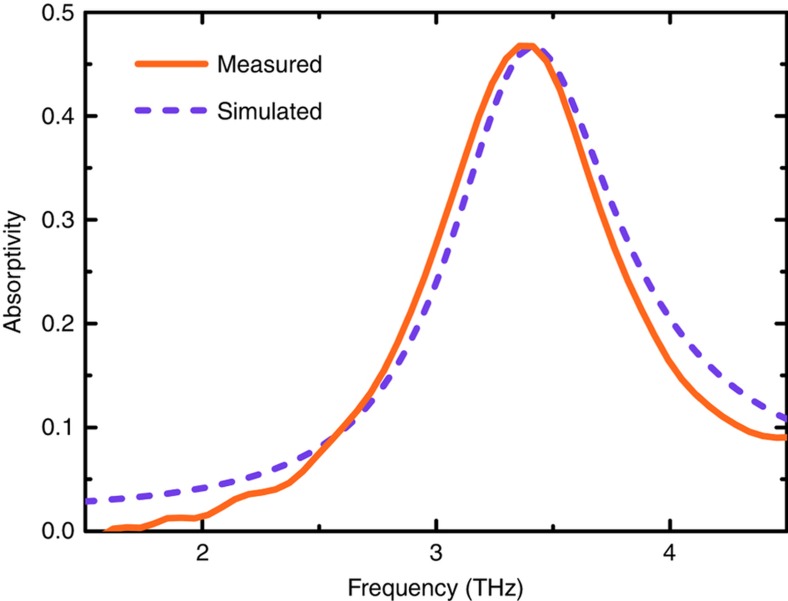
Measured and simulated absorption spectra of the meta-atom absorber in the THz regime.

The simulated and measured absorption spectra of the meta-atom absorber in the THz regime are plotted in Figure 4. An absorption peak, resulting from a dipole resonance (Supplementary Information), with a measured absorptivity of 0.47 at 3.40 THz can be observed. The high efficiency absorption of the THz wave provided by the meta-atom established the foundation for THz imaging for the meta-molecule. By altering the geometric structure of the meta-atom, especially that of the top resonator, multiband and broadband THz detections were easily realized.

To evaluate the noise limit, the theoretical NEP of the photomechanical meta-molecule was calculated to be 6.9 pW Hz^−1/2^ at 3.11 THz, which is the operating frequency of the THz source used for the following active imaging (Supplementary Information). The experimental NEP value of the entire imaging system, including the noise from the meta-molecule, vacuum system, optical readout system and THz source, was measured to be 2.9 nW Hz^−1/2^ at the same frequency. The three orders of difference between the theoretical and experimental NEP indicated that the noise was primarily attributable to the readout system. An external vibration might induce a larger noise, and an anti-vibration design for the vacuum packaging and sampling readout system would substantially decrease the noise. Other possible methods of decreasing the readout noise include using a more stable light source and a low-noise CCD of the readout system.

**Figure 5:**
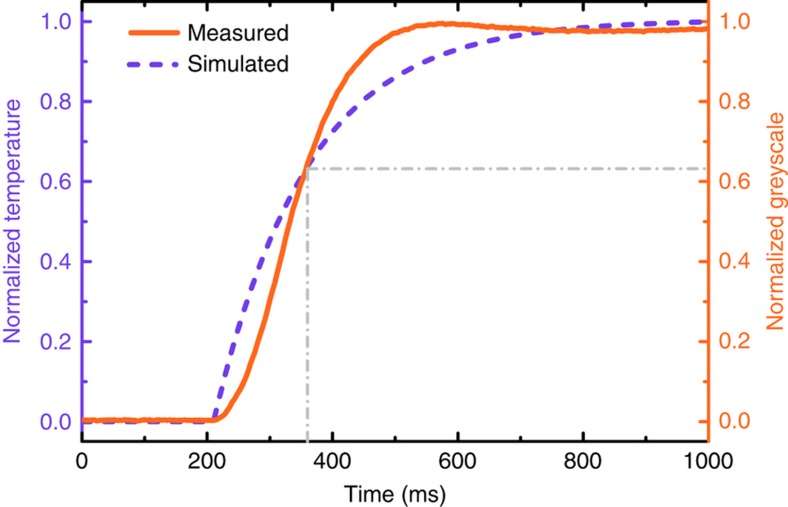
Measured and simulated transient response of the photomechanical meta-molecule in the time domain. The transient normalized temperature change of one meta-molecule in the time domain was simulated, and the transient normalized greyscale in the time domain was experimentally extracted from a series of captured THz images.

The response time is critical for estimating the real-time response of a detector. According to the definition, we simulated the transient temperature and measured the transient greyscale of one meta-molecule in the time domain to obtain the response time, as plotted in Figure 5. The measured response time of 140 ms was consistent with the simulated value of 150 ms (Supplementary Information), and this value ensures that the photomechanical meta-molecule array is capable of real-time imaging.

Additional factors that may influence the performance of the meta-molecule array are the external mechanical acceleration and the temperature cross-sensitivity. As a free standing structure, the meta-molecules can bear a vibration under a strong external acceleration but return to the equilibrium state soon after the external acceleration. The structure remains intact with consistent mechanical properties, and the sensitivity should not be affected before or after the acceleration. In practical settings, the anti-vibration design and packaging techniques can be applied to the array and the imaging system to eliminate the influence of the vibration.

For the temperature cross-sensitivity, the THz and infrared detection of the meta-molecule depend on the photonic-energy-caused temperature difference between the target and the environment. The change in the environment temperature affects only the initial position of all of the meta-molecules together, and its influence on the temperature difference is slight. Therefore, the temperature cross-sensitivity can be fundamentally suppressed by the proposed device. Additionally, for the active THz imaging in our case, the temperature difference in the meta-molecule primarily originates from the THz laser, and the THz emission from the non-target objects at room temperature can be negligible.

### Real-time imaging

**Figure 6:**
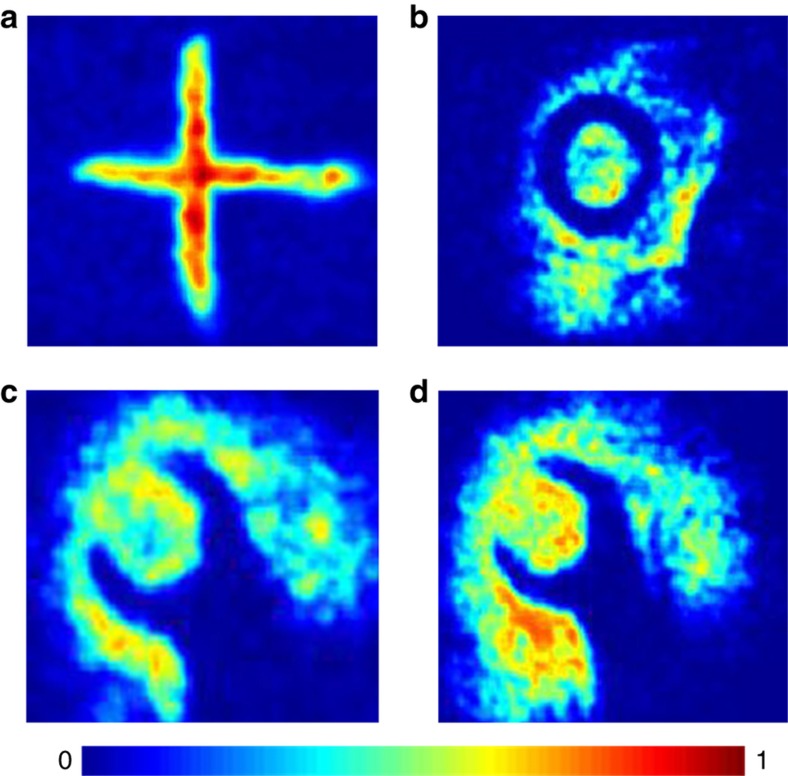
THz images of the metal samples obtained by the photomechanical meta-molecule array at room temperature. The images of a metal plate with a cross slit (**a**) and metal circle washer (**b**). The image of a metal wrench with (**c**) and without (**d**) covering the optically opaque HDPE plate. The color scale is normalized, and (**c**) and (**d**) share the same color range.

By sealing the fabricated photomechanical meta-molecule array in a vacuum chamber, the THz images of several objects were actively captured in transmission mode at room temperature by using the real-time imaging system. Figures 6a and b illustrate the THz images of a metal plate with a cross slit and a metallic circular washer, respectively. Owing to the THz opaqueness of the metallic material, a bright cross shape and a dark circle shape can be clearly observed in the two THz images. Because a variety of commonly used packaging materials are transparent to THz waves, we concealed a metal wrench behind an optically opaque HDPE plate with a thickness of 5 mm and captured the THz images of the same wrench with and without the blocked HDPE, as shown in Figures 6c and d. In spite of the energy attenuation caused by the HDPE plate (Supplementary Information), the THz wave penetrated through the plate and fully recorded the geometric features of the hidden wrench. The optical photos of the three metal objects and HDPE plate are provided in Supplementary Figure S7.

**Figure 7:**
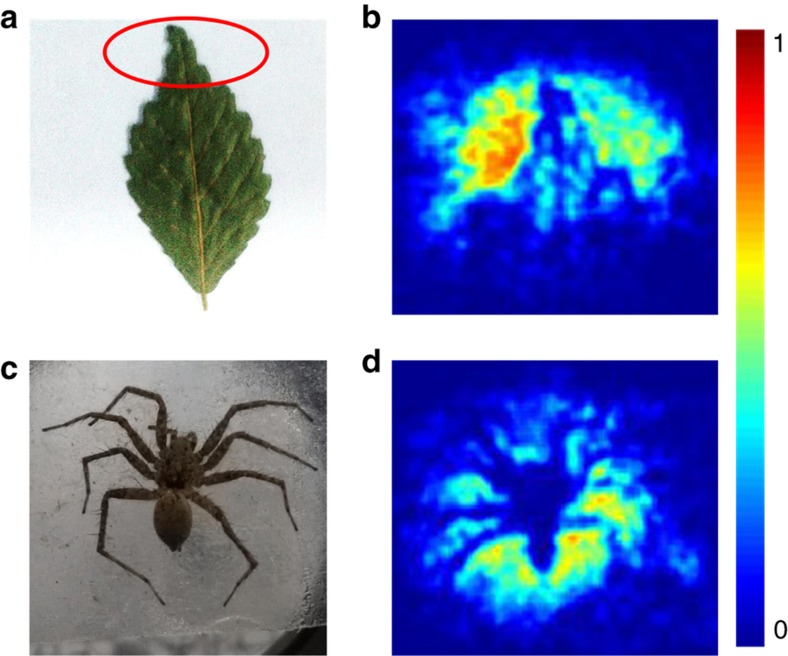
THz images of the biological samples obtained with the photomechanical meta-molecule array at room temperature. The optical photo of a dehydrated leaf with the apex marked by a red circle (**a**) and the THz image of the leaf apex (**b**). The optical (**c**) and THz images (**d**) of a fresh spider. The color scale is normalized.

Furthermore, the THz images of several biological samples were obtained by the fabricated meta-molecule array, and Figure 7 presents the THz images of the top part of a dehydrated leaf and a fresh spider. Unlike the metal sample, the THz wave can easily transmitted through the leaf, owing to its lack of water, and the apex and zigzag margin of the leaf are evident, owing to the scattering of the THz energy on the boundary. Additionally, the main and branching veins of the leaf can be clearly identified in the THz photo because of the difference in the THz transmittance in those regions. For the fresh spider sample, as a result of the rich water content of its body, the THz radiation was unable to transmit through the insect, and certain fine features, including its eight legs and tail, can be observed clearly. Our device’s ability to distinguish between these small energy variations caused by the leaf veins and spider legs demonstrates its high sensitivity and resolution.

To demonstrate the real-time imaging ability of the photomechanical meta-molecule array, a THz video recording of a moving metal wrench is shown in supplementary video with both the acquisition and play frame rates at 20 Hz. The static and dynamic THz imaging results demonstrated proof of concept for the proposed photomechanical meta-molecule array and indicated that it can be applied in actual cases at a video rate.

The measured NEP and the acquired THz images demonstrated that the sensitivity of our device is competitive with those of commercially available uncooled THz detectors at room temperature^10^. The state-of-the-art complementary metal oxide semiconductor (CMOS)-based THz detectors present average NEPs similar to those of our meta-molecule array^15,32^; however, the requirements of the nanoscale line width and fabrication techniques cause their detection of high frequency THz waves to be fundamentally challenging^10^. The carbon-nanotube (CNT) THz detectors present a relatively faster response time; however, their NEPs are on the order of ~10 nW Hz^−1/2^ (Refs. 13,33). Certain graphene-based THz detectors indicate a considerably lower NEP with a fast response time^34,35^; however, the immaturity of the graphene and CNT fabrication techniques severely limits the practical application of the carbon-based THz detectors in the short term. More importantly, these newly reported THz detectors are single pixel or in linear array, which have the drawbacks of either long image acquisition times due to raster scanning or low image resolution due to the design and fabrication complexity. Because of its non-electric design, upgrading our meta-molecule array to a higher resolution should be very easy.

**Figure 8:**
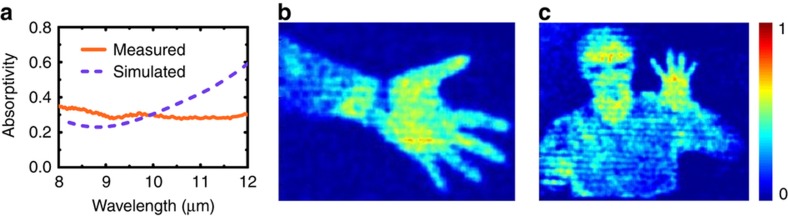
Passive infrared imaging of the photomechanical meta-molecule array. The simulated and measured absorption spectra of the meta-atom in the infrared regime (**a**). A man with a watch on his wrist (**b**) and a half-length portrait (**c**) show clearly identifiable details due to the temperature difference. The color scale is normalized.

As indicated above, the SiN_x_ structural layer of the meta-atom absorbers demonstrates a high absorption in infrared regions^36^. Therefore, we also assessed the capability of our fabricated device for use in passive infrared imaging at room temperature with methods and systems similar to those used for the THz wave. The absorption characteristics of the meta-atom absorber in the infrared regime were first studied, and the simulated and measured absorption spectra of the meta-atom absorber in the infrared regime are illustrated in Figure 8a. The simulation was accomplished by using COMSOL Multiphysics, and the Au was characterized as a Drude metal with a plasma frequency of 2π×2175 THz and a collision frequency of 2π×6.5 THz (Ref. 37), and the SiN_x_ was modeled on the basis of the complex permittivity obtained from Ref. 38. The measurements were performed with a microscope-coupled FTIR spectrometer (magna-IR 750) in ambient pressure at room temperature. As observed from Figure 8a, between the wavelengths of 8 and 12 μm, a flat band absorption resulting from the SiN_x_ spacer is obtained with an average absorptivity of 0.30, thus allowing the meta-molecule to sense the infrared wave. In the infrared regime, the theoretical NEP was calculated to be 9.2 pW Hz^−1/2^, and the response time was the same as the characterized value in the THz regime, owing to its independence from the power of the incident wave and the absorptivity of the meta-atom absorber (Supplementary Information).

In the passive infrared imaging system, the material used for the lenses and chamber window is germanium coated with an anti-reflection film (Supplementary Information). As indicated in Figures 8b and c, infrared images of a human hand and a half-length portrait were captured by using the same photomechanical meta-molecule array and showed distinct details, such as the watch, glasses and shirt collar. A brighter color was observed at the palm, forehead and neck because of a slightly higher temperature, thus indicating that the photomechanical meta-molecule array is capable of imaging the infrared radiation with high sensitivity.

Owing to the artificially tunable properties of the meta-atom absorber, the proposed meta-molecule array can be conveniently configured to image at different THz frequencies as well as multiband and broadband THz waves. Specifically, we believe that the simultaneous and real-time imaging abilities of the THz and infrared by using the same device would be widely favored in both scientific and commercial fields because their properties and advances are complementary in several aspects. The infrared imaging technique surpasses the THz imaging method in sensing small differences in temperature, whereas the THz wave exceeds the infrared wave in identifying concealed objects with uniform temperature distribution.

## Conclusions

In conclusion, we designed, fabricated and characterized a novel meta-molecule array with 128×128 pixels for uncooled real-time THz imaging based on photomechanical effects. Through the introduction of a layer of periodic cross resonators on top of the SiN_x_ structural layer, in which the bottom gold mirror functions as the ground plane, the meta-atom absorbers were simply assembled with thermal-sensitive bi-material cantilevers. A glass wafer was used to support the meta-molecule array with the readout light transmitting through it, thus completely avoiding the THz energy loss from the substrate with the THz wave incident from the front side. Benefiting from the advantages of the meta-atom absorber, the meta-molecule array successfully acquired THz images of metal and biological objects in real time and was able to record a THz video at a frame rate of 20 Hz. The NEP of the imaging system was measured to be 2.9 nW Hz^−1/2^ in the THz regime. Additionally, infrared photos of the human body were captured by using the infrared absorption characteristics of the SiN_x_ dielectric spacer and showed several distinct details. Our approach for real-time THz and infrared imaging presents fascinating practical values in a wide scope of applications, such as security monitoring, non-destructive inspections and medical diagnoses.
