# Supplementary material for: Photomechanical meta-molecule array for real-time terahertz imaging
Source: Microsyst Nanoeng. 2017 Dec 4;3:17071. doi: 10.1038/micronano.2017.71 (PMC6444985; doi:10.1038/micronano.2017.71)
Supplement: Supplementary Information [file micronano201771-s1.pdf]

Supplementary file

Photomechanical meta-molecule array for real-time terahertz imaging

Yongzheng Wen[1,2], Delin Jia[1], Wei Ma[1], Yun Feng[3], Ming Liu[3], Liquan Dong[3], Yuejin Zhao[3] and Xiaomei Yu[1]

Microsystems & Nanoengineering (2017) **4,** 17071; doi:10.1038/micronano.2017.71; Published online: 4 December 2017

I. DESIGN THEORY OF THE META-MOLECULE

The most critical characteristics for a detector are responsivity, noise and response time. When THz radiation is absorbed by the meta-atom absorber, the temperature of the meta-molecule increases according to[1]:

$$\Delta T = \frac{\eta P_0}{G_{\text{total}}\sqrt{1+\omega^2\tau^2}}, \tag{1}$$

where P_0 is the incident THz power on a meta-molecule, η is the THz absorptivity of the meta-atom absorber, ω is the modulation frequency of the incident THz wave, G_{total} is the total thermal conductance of the meta-molecule, and τ is the response time. The responsivity, R_v, is defined as the displacement of the meta-molecule tip, Δz, produced in response a given incident THz radiation power falling on one meta-molecule, which can be expressed as[2]:

$$R_v = \frac{\Delta z}{P_0} = \eta\left(\frac{\Delta z}{\Delta T}\right)\frac{1}{G_{total}\sqrt{1+\omega^2\tau^2}} = \eta\frac{S_T}{G_{total}\sqrt{1+\omega^2\tau^2}}, \tag{2}$$

where S_T stands for the thermomechanical sensitivity, used to evaluate the displacement of the meta-molecule tip in response to the temperature change ΔT. Since in the active imaging system, the THz wave radiates from a laser with the output power density of P_t and transmits through the imaging optics before it reaches the photomechanical meta-molecule array, it is obvious that the imaging optics should be considered in the responsivity. In that case, the THz power falling on one meta-molecule, P_0, can be obtained by:

$$P_0 = \tau_0\frac{A_d}{4F^2}P_t, \tag{3}$$

where τ_0 is the transmissivity of the THz optics, F is the f-number of the optics, A_d is the absorption area of the meta-molecule. By combing the supplementary equations (2) and (3), the responsivity to the THz laser, R_L can be obtained as:

$$R_L = \frac{\Delta z}{P_t} = \frac{A_d\tau_0\eta S_T}{4F^2 G_{total}\sqrt{1+\omega^2\tau^2}}. \tag{4}$$

It is clear that with constant parameters of the THz optics, large absorption area of the meta-molecule will improve the responsivity. Limited by the chip size and array scale, the area of the meta-atom absorbers was designed as 180 μm × 100 μm. The supplementary equation (4) also indicates that besides the absorption area, the high THz absorptivity, high thermomechanical sensitivity and low thermal conductance will benefit the responsivity. As the absorptivity is determined by the meta-atom absorber, the other two factors should also be considered in the design and optimization of the proposed meta-molecule.

The thermomechanical sensitivity can be expressed as[3]:

$$S_T = \frac{\Delta z}{\Delta T} = 3\ (\alpha_1 \quad \alpha_2)\frac{L_{bi}^2}{t_1+t_2}\left[\frac{(1+\frac{t_1}{t_2})^2}{3(1+\frac{t_1}{t_2})^2+(1+\frac{t_1E_1}{t_2E_2})(\frac{t_1^2}{t_2^2}+\frac{t_2E_2}{t_1E_1})}\right]. \tag{5}$$

where L_{bi} is the length of the bi-material cantilever, t_1 and t_2, E_1 and E_2, and α_1 and α_2 are the thicknesses, Young's Moduli, and thermal coefficients of expansion (TCE) of the two materials respectively. As indicated in the equation, the difference in TCE, the ratio of the thicknesses, and the length of the bi-material cantilever dominate its thermomechanical sensitivity. Al and SiN_x were used as the composite materials since their difference in TCE is big. The relation of the thermomechanical sensitivity versus the Al/SiN_x thickness ratio and length of the bi-material cantilever was calculated. As plotted in Supplementary Figure S1, S_T reaches more than 94% of its maximum value with the thickness ratio more than 0.5, and the highest sensitivity is achieved at the ratio of 0.68. Since a thickness of 800 nm for SiN_x layer is optimized for the meta-atom absorber to obtain high THz absorption, a 400 nm thick Al film was deposited as the top layer of the bi-material cantilevers. This thickness brings great convenience in the fabrication as well as high S_T. Supplementary Figure S1 also shows the dramatic enhancement on thermomechanical sensitivity as the cantilever length increased from 100 to 200 μm, so the length of the bi-material cantilever needs to be lengthened.

On the other hand, the thermomechanical sensitivity can also be expressed with the relation of the angular deflection and the temperature change as[3]:

$$S_{T\theta} = \frac{\Delta\theta}{\Delta T} = 6\ (\alpha_1 \quad \alpha_2)\frac{L_{bi}}{t_1+t_2}\left[\frac{(1+\frac{t_1}{t_2})^2}{3(1+\frac{t_1}{t_2})^2+(1+\frac{t_1E_1}{t_2E_2})(\frac{t_1^2}{t_2^2}+\frac{t_2E_2}{t_1E_1})}\right]. \tag{6}$$

By combing with the supplementary equation (1), the responsivity with the angular deflection can be obtained as:

$$R_{v\theta} = \frac{\Delta\theta}{P_0} = \frac{\eta S_{T\theta}}{G_{total}\sqrt{1+\omega^2\tau^2}}. \tag{7}$$

The total thermal dissipation of the meta-molecule mainly originates from the thermal isolation cantilevers, the radiation and air flow. As most area of the meta-molecule is covered with metal, whose emissivity is quite small, the radiative component of the thermal loss can be neglected. Under the vacuum pressure,

Q1 [1]National Key Laboratory of Science and Technology on Micro/Nano Fabrication, Institute of Microelectronics, Peking University, Beijing 100871, China; [2]State Key Laboratory of New Ceramics and Fine Processing, School of Materials Science and Engineering, Tsinghua University, Beijing 100084, China and [3]Beijing Key Laboratory for Precision Optoelectronic Measurement Instrument and Technology, School of optoelectronic, Beijing Institute of Technology, Beijing 100081, China
Correspondence: Yuejin Zhao or Xiaomei Yu (yjzhao@bit.edu.cn or yuxm@pku.edu.cn)

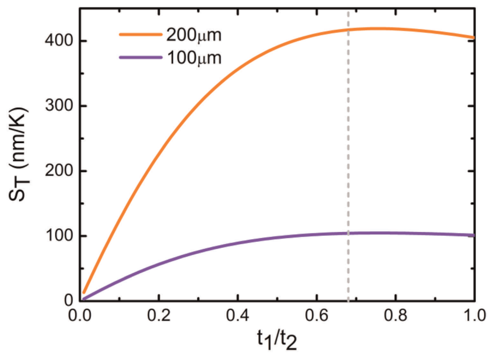

Figure 1 Calculated thermomechanical sensitivity versus the thickness ratio of Al/SiN$_x$ for the bi-material cantilever. The length of the bi-material cantilever is selected to be 100 and 200 µm, and the maximum values are marked with grey dashed line.

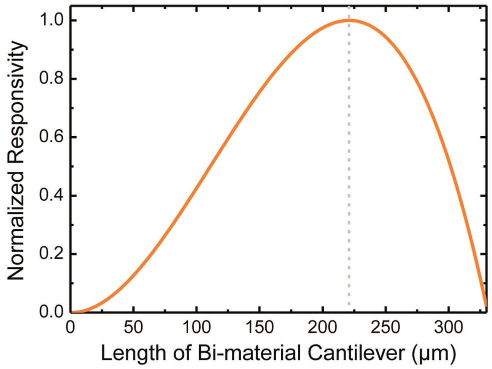

Figure 2 Calculated normalized responsivity versus the length of the bi-material cantilever. In the calculation, the other geometric and material parameters are kept constant, and the maximum values are marked with grey dashed line.

the thermal dissipation through air is minimized and can also be eliminated. The total thermal conductance of the meta-molecule is hence simplified as:

$$G_{leg} = 2\frac{A_{iso}k_{SiNx}}{L_{iso}}, \quad (8)$$

where k_{SiNx} is the thermal conductivity of the SiN$_x$, and L_{iso} and A_{iso} are the length and cross-sectional area of the isolation cantilevers, respectively. The thickness of the isolation cantilevers is consistent with that of the SiN$_x$ structural layer, and its width is required to be wide enough to support the meta-molecule, which is designed as 4 µm. In that case, increasing the length of the isolation cantilevers becomes the main approach to reduce thermal conductance of the meta-molecule.

Considering the supplementary equations (5) and (8), both the thermomechanical sensitivity and thermal conductance desire long cantilevers, whereas the unlimited extension of their lengths is clearly unreasonable. In fact, overlong bi-material cantilevers will cause large intrinsic deflection of the meta-molecule due to the residual stress mismatch from the fabrication, which brings much trouble in imaging. As will be described below, low thermal conductance, originated from long isolation cantilevers, provides high responsivity but slow response speed. In addition, overlong cantilevers will result in low fill factor of the meta-molecule, leading to low quality of the acquired images. As a result, by taking the above factors into account, the total length of bi-material and isolation cantilevers is designed as 330 µm, which is approximately twice as long as the length of the absorber. By combining the supplementary equations (2), (5) and (8), the relation, $R_v \propto L_{bi}^2 \times L_{iso}$, can be obtained by keeping other parameters constant. Given $L_{bi}+L_{iso} = 330$ µm, the relation is evolved as $R_v \propto L_{bi}^2 \times (330\text{-}L_{bi})$, which is calculated and plotted in Supplementary Figure S2 with the responsivity normalized. The responsivity reaches more than 96% of its maximum with the length of the bi-material cantilever ranging from 195 to 245 µm. Although the optimum value is achieved at the length of 221 µm, it results in relatively short isolation cantilever and high thermal conductance, which is undesired in minimizing the noise. Therefore, the designed bi-material cantilevers were optimized as 209 µm, and the corresponding length of the isolation cantilevers were 133 µm.

In that case, the total thermal conductance, and the thermomechanical sensitivity with the displacement and the angle can be calculated as 2.65×10^{-7} W/K, 426.5 nm/K, and 0.234 deg/K respectively. With the supplementary equations (2) and (7), the responsivity with the displacement and the angle can then be obtained as 639.8 mm/W and 3.5×10^5 deg/W, respectively.

Noise equivalent power (*NEP*) characterizes the sensitivity of the detector, which is defined as the signal power that gives a signal-to-noise ratio of unity in a one hertz output bandwidth. There are three primary noise sources in our device: (1) the thermal fluctuation noise due to continuous heat exchange (NEP_{TF}); (2) the background fluctuation noise due to radiative heat exchange with the environment (NEP_{BF}); (3) the thermomechanical noise due to thermally driven random motion of the mechanical structure (NEP_{TM})[2]. These three *NEPs* can be expressed by:

$$NEP_{TF} = \frac{T_p\sqrt{4k_BBG_{total}}}{\eta}, \quad (9a)$$

$$NEP_{BF} = \frac{\sqrt{16k_BA_{meta}B\sigma_T\left(T_b^5 + T_p^5\right)}}{\eta^{1/2}}, \quad (9b)$$

$$NEP_{TM} = \frac{1}{R_v}\sqrt{\frac{4k_BT_pB}{Qk\omega_0}}, \quad (9c)$$

where T_p is the temperature of the meta-molecule, T_b is the background temperature, k_B is the Boltzmann constant, B is the measurement bandwidth, A_{meta} is the area of the meta-molecule, σ_T is the Stefan-Boltzmann constant, Q is the quality factor of the meta-molecule, ω_0 is the mechanical frequency of the meta-molecule, and k is the spring constant of the meta-molecule. Using the eigenfrequency solver in the COMSOL structural mechanics module, the first resonance and spring constant of the meta-molecule were simulated as 1093 Hz and 0.003 N/m, respectively. The total *NEP* can thus be obtained by:

$$NEP = \sqrt{NEP_{TF}^2 + NEP_{BF}^2 + NEP_{TM}^2}. \quad (10)$$

Supplementary equations (9) and (10) indicate that the *NEP* can be decreased by improving the absorptivity and responsivity, and decreasing the thermal conductance. As mentioned above, the optimization of the bi-material and isolation cantilevers makes the responsivity very close to the optimum value, and low thermal conductance is also realized.

The NEP is thus calculated as 6.9 pW/Hz$^{1/2}$ at the working frequency of 3.11 THz. With the definition equation of NEP, $NEP = z_n/R_v = \theta_n/R_{v\theta}$[2,4,5], the noise-caused displacement (z_n) and angle (θ_n) of 4.4 pm and 2.42 µdeg could be calculated respectively. Considering 1 mW THz wave incident on the whole meta-molecule array, the power falls on one meta-molecule is 61.0 nW, and the signal-caused displacement and angle are respectively 39 nm and 0.021 deg, which are much more evident than the noise signal and present high sensitivity of the meta-molecule.

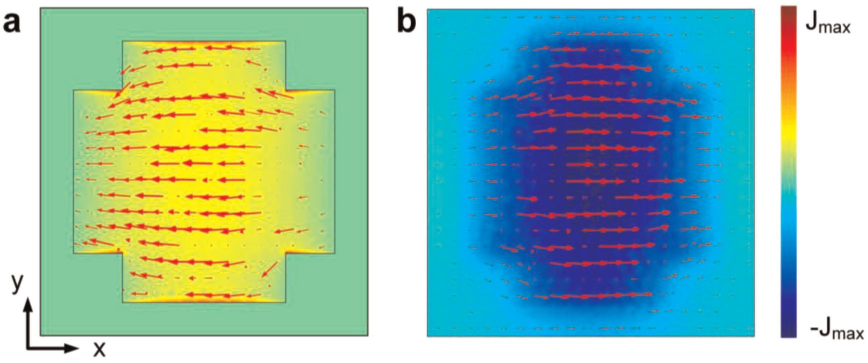

Figure 3 Simulated surface currents distribution of the meta-atom absorber at 3.11 THz. The surface currents distribution in the cross resonator (**a**) and in the ground plane (**b**). The color map represents the amplitude of the *x* component of the surface currents.

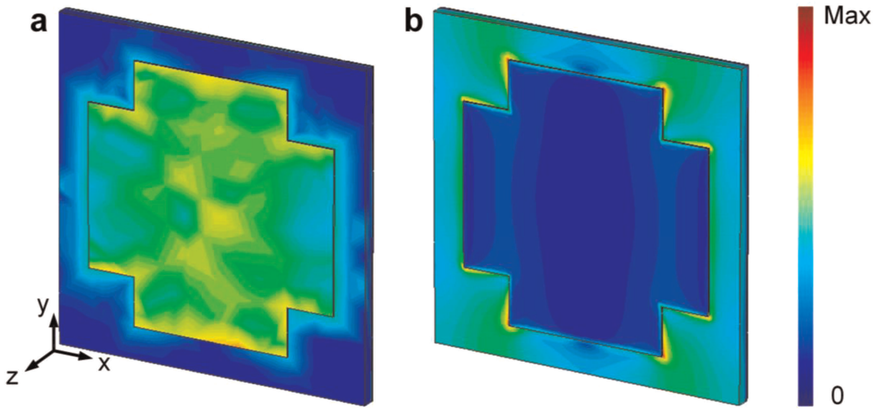

Figure 4 Simulated power loss distribution of the meta-atom absorber. The power loss distribution at frequency of 3.11 THz (**a**) and wavelength of 10 μm (**b**).

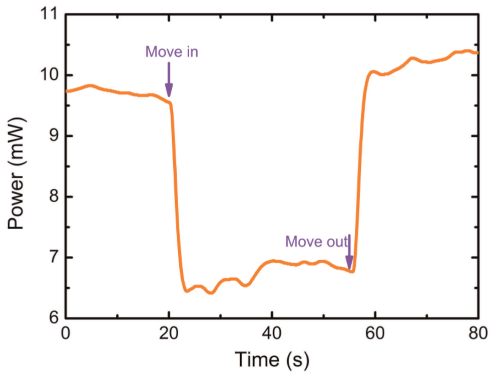

Figure 5 THz power response with a HDPE plate to block the radiation. The 5 mm thick HDPE plate was inserted between the THz source and power meter, and the moments of moving in and out are marked in the figure.

The response time of the device is also a crucial figure of merit to evaluate the speed of the response, which can be obtained by:

$$\tau = \frac{C_{th}}{G_{total}} = \frac{\rho V c}{G_{total}}, \tag{11}$$

where C_{th} is the total heat capacitance of the device, ρ is the density, V is the volume, and c is the specific heat capacity. Supplementary equations (2) and (8)-(11) show that low thermal conductance provides high responsivity and low *NEP*, but slows down the response speed. Consequently, a tradeoff between these figures of merit needs to be generally considered in the design of the meta-molecule, especially the length of isolation cantilevers. Meanwhile, it is worth noting that for given materials and thermal conductance, an effective method to improve the response time is to reduce the volume of the meta-molecule. In our design, the response time was calculated as 124 ms.

II. DESIGN AND PRINCIPLE OF THE META-ATOM ABSORBER

There are four main considerations in the design of the meta-atom absorber. Firstly, the resonator should response to the THz wave at target frequency and be insensitive to the polarization in order to suit different THz source. Secondly, the thickness of the dielectric spacer should be as thin as possible on the condition that the absorptivity of the THz wave is reasonably high, which are both strongly desired in the achievement of high sensitivity in THz imaging. Thirdly, it is required that the release holes are embedded in the meta-atoms with little sacrifice in absorption. At last, all the geometric constants ought to be designed to satisfy the requirement of accuracy provided by the standard photo-lithography. With adequate consideration of the four necessities, the final design of the meta-atom absorber is shown in Figure 1 in the main body of the article with the thickness of the dielectric spacer as 800 nm and the dimension of the release holes as 7 μm × 7 μm.

To understand the origin of the THz and infrared absorption, we simulated the surface currents and power loss distribution of one meta-atom using COMSOL Multiphysics, and a plane wave is assumed to illuminate in *z* direction with the electric field along *x* axis. In THz regime, at the working frequency of 3.11 THz, the

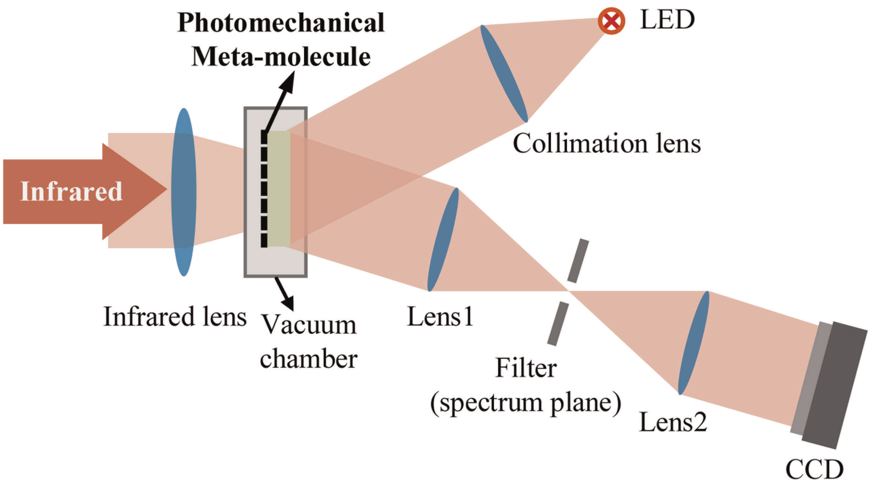

Figure 6 Supplementary Figure S6Schematic of the passive infrared imaging system.

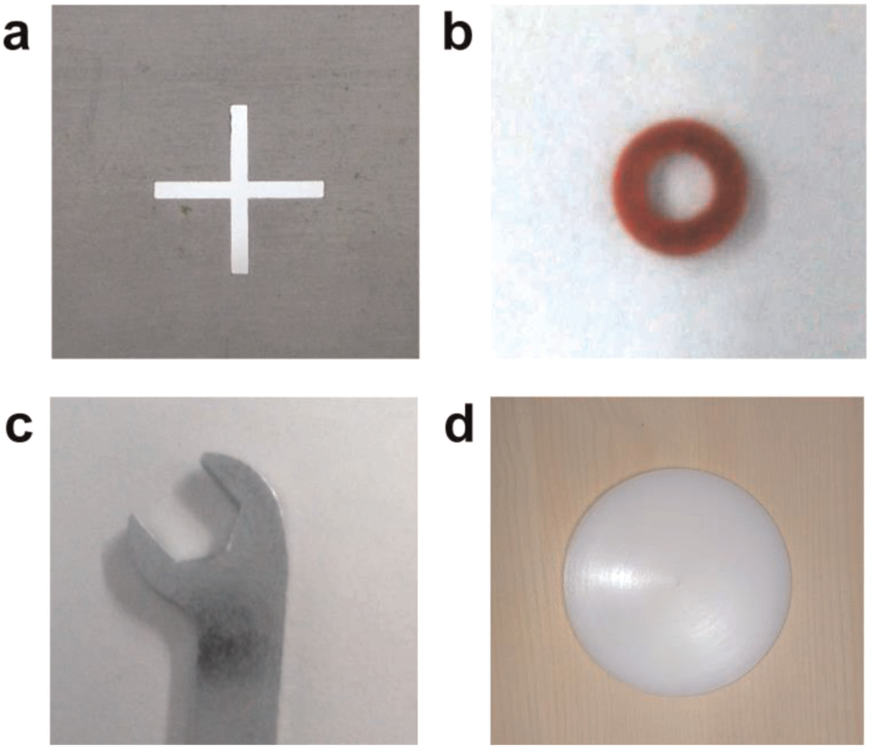

Figure 7 Optical photos of the objects for THz imaging. The metal plate with a cross slit (**a**), the metal circle washer (**b**), the metal wrench (**c**), and the HDPE plate for concealing the wrench (**d**).

Table 1 The physical parameters of the materials used for the photomechanical meta-molecule array[3]

Material	*Young's Modulus (10^9 Pa)*	*Thermal Expansion Coefficient ($10^{-6}K^{-1}$)*	*Thermal Conductivity (W/m·K)*	*Specific Heat Capacity (J/kg·K)*	*Density (kg/m^3)*
SiN_x	180	2.1	5.5	691	2.4
SiO_2	68	0.4	1.4	703	2.2
Al	70	25	237	900	2.7
Au	77	14.2	296	129	19.3

simulated surface currents distribution in the cross resonator exhibits a typical dipole resonance with anti-parallel currents in the ground plane, as revealed in Supplementary Figure S3. The dipole resonance in the cross resonator couples strongly with the electric filed of the THz wave, while its magnetic field resonates with the cross resonator and the ground plane, thus producing the anti-parallel currents[6]. By tuning each of the resonances, it is possible to match the impedance of the meta-atom absorber to free space and minimize the reflectance (*R*)[7]. Along with the block of THz transmission (*T*) by the metal ground plane, a high absorption (*A*) is achieved (*A* = *1-T-R*). The simulated power loss distribution, as revealed in Supplementary Figure S4(a), shows the primary loss occurs in the dielectric spacer beneath the cross resonator, which also supports that the THz absorption originated from the coupling between the meta-atom and the incident THz wave.

The simulated power loss distribution at the wavelength of 10 μm is depicted in Supplementary Figure S4(b). Since the geometric parameters of the cross are larger than the wavelength, the metal cross layer does not behave as a resonator, and no resonance peak was observed. Therefore, the loss mainly exists in the region of the SiN_x spacer not covered by the gold film, which demonstrates that the infrared radiation is absorbed by the exposed SiN_x film rather than the meta-atom absorber structure.

III. THZ ENERGY ATTENUATION OF THE HDPE PLATE

A 5 mm thick HDPE plate was used as the THz window of the vacuum chamber, and another one was also used to conceal the metal wrench in the imaging experiment. Although the HDPE material offers high transmission in THz regime, it still causes the non-negligible THz energy attenuation due to the relatively large thickness. We recorded the THz power change in the process of moving the HDPE plate in and out by a THz power meter (Ophir Nova II with 3A-P-THz), and the same THz source (OPTL) was used. As plotted in Supplementary Figure S5, with the HDPE plate pushed in between the THz source and power meter, the detected average power drops from 9.7 to 6.9 mW with the transmissivity of 0.71. The instability of the output power is mainly originated from the THz source.

IV. INFRARED IMAGING SETUP AND CHARACTERISTICS

The schematic diagram for the infrared imaging is depicted in Supplementary Figure S6. Different from the THz imaging, the infrared imaging is passive without any external infrared source. As mentioned in the main text, the material used for the infrared lenses and chamber window is germanium with antireflection film coated. The rest parts of the infrared imaging setup, including the vacuum chamber, the optical readout and the CCD, are the same as those in the THz system.

As described in the main text and analyzed above, the capability of the meta-molecule array sensing the infrared wave mainly originates from the infrared absorption of the SiN_x layer rather than the whole meta-atom absorber. Despite that, the infrared imaging shares the same mechanical and thermal characteristics with the THz one, such as the thermomechanical sensitivity, the thermal conductance and capacitance, spring constant, and the response time. With the measured average absorptivity of 0.30 within the wavelength from 8 to 12 μm, the NEP was calculated as 9.2 $pW/Hz^{1/2}$, corresponding to the noise-caused displacement of 5.9 pm.

For the infrared imaging, the temperature change of the meta-molecule ΔT_{meta} due to the target infrared source ΔT_{IR} can be expressed as[3]

$$H = \frac{\Delta T_{meta}}{\Delta T_{IR}} = \frac{A_d \tau_0 \eta \pi}{4F^2 G_{total}} \cdot \left(\frac{dP}{dT_t}\right), \tag{10}$$

where dP/dT_t is the fraction of the radiative energy emitted by the target source at temperature T_t, which is 0.63 $W/(m^2{\cdot}sr{\cdot}K)$ within the 8 to 12 μm spectral band[3]. Taking the *F* number as 1 and the transmissivity τ_0 as 0.9 for the infrared optics, the *H* can be calculated as 0.0091, which means 1 K change of the target temperature would cause 9.1 mK variation of the meta-molecule. Given the temperature difference of 16 K between the human body (36 °C) and the room temperature (20 °C), and the thermomechanical sensitivity of 427 nm/K, the displacement of the meta-molecule tip was obtained as 62 nm, which is much larger than the noise signal.

REFERENCES

1 Kruse PW, Skatrud DD. Uncooled infrared imaging arrays and systems. in. *Uncooled Infrared Imaging Arrays and Systems* 1997; **1**.

2 Datskos PG, Lavrik NV, Rajic S. Performance of uncooled microcantilever thermal detectors. *Review Of Scientific Instruments.* 2004; **75**: 1134.

3 Zhao Y *et al.* Optomechanical uncooled infrared imaging system: Design, microfabrication, and performance. (in English). *Journal Of Microelectromechanical Systems, Article* 2002; **11**: 136–146.

4 Kwon B, Rosenberger M, Bhargava R *et al.* Dynamic thermomechanical response of bimaterial microcantilevers to periodic heating by infrared radiation. (in eng). *Review Of Scientific Instruments, Research Support, U.S. Gov't, Non-P.H.S* 2012; **83**: 015003.

5 Schäffer. TELow-Noise Methods for Optical Measurements of Cantilever Deflectionsin. *Applied Scanning Probe Methods V*. Springer. 2007, 51–74.

6 Landy N, Sajuyigbe S, Mock J *et al.* Perfect Metamaterial Absorber. *Physical Review Letters.* 2008; **100**: 207402.

7 Hu T, Landy NI, Bingham CM *et al.* A metamaterial absorber for the terahertz regime: design, fabrication and characterization. *Optics Express.* 2008; **16**.
